# Short-term effects of atropine on the retina and choroid in young adults

**DOI:** 10.1186/s40662-026-00477-1

**Published:** 2026-03-01

**Authors:** Barsha Lal, Lisa A. Ostrin

**Affiliations:** University of Houston College of Optometry, 4401 Martin Luther King Blvd, Houston, TX 77004 USA

**Keywords:** Atropine, Axial length, Choroid, Myopia, OCT imaging

## Abstract

**Background:**

Low concentration atropine is widely prescribed to slow myopia progression in children, yet its short-term retinal and choroidal effects remain incompletely understood. This study aimed to evaluate short-term effects of a range of low atropine concentrations on axial length, retinal and choroidal thickness, and microvasculature.

**Methods:**

In this double-masked, randomized study, twenty healthy adults received a single instillation of placebo, 0.01%, 0.025%, 0.05%, or 0.1% atropine in the right eye across five separate sessions. Retinal and choroidal thickness in the central 1.0 mm diameter and 1.0–3.0 mm annulus, perfusion density in the superficial and deep vascular complex and choriocapillaris in the central 1.0 mm and 1.0–2.5 mm annulus, foveal avascular zone and axial length were assessed at baseline and 1 h and 24 h after instillation.

**Results:**

Participant mean age was 25.5 ± 3.4 years and mean refraction was − 1.9 ± 2.2 D. No significant changes in retinal or choroidal thickness or axial length were observed for any concentration or time point (*P* > 0.05). The superficial vascular plexus perfusion density in the 1.0–2.5 mm annulus showed significant decrease (*P* = 0.02) with time after atropine instillation, but not with concentration (*P* > 0.05); post hoc analysis showed significant decrease from baseline at 1 h (*P* = 0.03) compared to 24 h (*P* = 0.28).

**Conclusion:**

These findings indicate that a single instillation of 0.01%–0.1% atropine does not alter axial length or retinal or choroidal thickness over 24 h, but may transiently affect superficial retinal perfusion in a time-dependent manner. Characterizing these short-term effects is important for a better understanding of the physiological responses to atropine in clinical and research settings.

## Background

Myopia is projected to contribute to 27%–43% of uncorrectable visual impairment by 2050 in the United States [1]. On a global scale, the prevalence of myopia is currently estimated to be approximately 34% and is expected to increase to 50% by 2050 [2]. A 10% increase in the prevalence of myopia is estimated to result in an additional one million cases of visual impairment, thereby exacerbating the public health burden [1]. Individuals with myopia are at an increased risk for developing sight-threatening ocular pathologies [3]. Consequently, interventions aimed at preventing or delaying the onset of myopia are likely to yield substantial public health benefits.

Current strategies for myopia control include optical, pharmacological, environmental, behavioral, and surgical interventions [4]. Among these strategies, atropine is the most widely used pharmacological treatment and has shown effectiveness for myopia control in clinical trials among children [5]. The only other pharmacological treatment is oral 7-methylxanthine that is limited to Denmark [6]. Atropine was initially introduced for myopia control due to its cycloplegic properties, which temporarily paralyze the ciliary muscle and thereby inhibit accommodation [7]. This approach was based on the early hypothesis that excessive or sustained accommodation contributed to the development and progression of myopia. Later, evidence from a chick model showed that atropine prevented experimental myopia via a non-accommodative mechanism [7, 8]; however, atropine’s mechanism of myopia control has not yet been elucidated.

Atropine is a nonspecific muscarinic receptor that inhibits all five types (M1–M5) of acetylcholine muscarinic receptors. Muscarinic receptors are widely distributed throughout the eye, including the iris (M3), ciliary body (M3), lens (M1 and M3), retina (M1, M2, and M3), and sclera (M1–M5) [9, 10]. Evidence from animal models suggest that muscarinic receptors are present in the choroid [7, 11]. Atropine has been shown to modulate biochemical signaling and alter scleral remodeling [12], which may contribute to its anti-myopia effects.

Various concentrations of atropine, ranging from 0.0025% to 1%, have been used for myopia control in children. Concentrations of 0.1% or less are considered “low concentration” [13]. A meta-analysis of 44 studies reported that both the efficacy and the risk of adverse events associated with atropine for myopia are largely dose-dependent, with higher concentrations generally resulting in greater efficacy and slower myopia progression, but accompanied by more severe side effects, including glare, photophobia, and blurring of near vision [14].

The choroid has been implicated as a potential mediator of atropine’s effects. Changes in choroidal thickness may promote the release of growth factors and improve microcirculation, thereby reducing scleral hypoxia and regulating extracellular matrix remodeling [7, 15]. Several studies in children and adults have reported choroidal thickening following atropine administration, observed both in the short term (1 h to 1 week) [16–19] and long term (6 months to 2 years) [17, 20]. The Low-concentration Atropine for Myopia Progression (LAMP) study found a dose dependent choroidal response to 0.05%, 0.025%, or 0.01% over 2 years, with a thicker choroid at higher atropine concentration [17]. On the contrary, other studies report no changes in choroidal thickness after 3 months to 2 years [17, 21] or choroidal thinning after 6 months of 0.01% atropine use [20]. Recent studies have also explored the effects of atropine on retinal and choroidal perfusion density as potential biomarker using optical coherence tomography angiography (OCTA). One study reported an increase in perfusion density after 3–6 months of 0.01% atropine. whereas another study found no changes in perfusion density [21].

While long-term effects of atropine on choroidal thickness have been relatively well studied, short-term effects remain relatively less explored. Evaluating early ocular changes may help better understand sites of action, refine dosing strategies, and predict long-term outcomes. Although meta-analyses suggest a dose–response relationship in long-term choroid changes, it remains unclear whether similar results occur in the short term. Additionally, examining retinal and choroidal microvasculature may provide deeper insight into atropine’s mechanisms of action. This study aimed to systematically investigate short-term effects of 0.01%, 0.025%, 0.05%, and 0.1% atropine on axial length, retinal and choroidal thickness, and the microvasculature in young adults.

## Methods

### Study design and ethics

This was a randomized, double masked, repeated measures study. Healthy adult participants between the ages of 18 and 35 years were recruited. Inclusion criteria were best-corrected visual acuity of 20/25 or better, noncycloplegic autorefraction (spherical component) between − 6.00 to + 3.00 D, astigmatism ≤ 2.00 D, objectively measured accommodative amplitude ≥ 3.00 D, normal ophthalmological and general health findings, and open anterior chamber angle. Exclusion criteria were narrow angles (Van Herick grade 2 or less), history of adverse reaction with any dilating eye drops, or any myopia control treatment. Participants underwent screening through a detailed medical and ocular history, visual acuity assessment, noncycloplegic autorefraction, and objective accommodation testing using an open-field autorefractometer (WAM 5500, Grand Seiko, Japan).

Written informed consent was obtained from all the participants after explaining the purpose and risks of the study. The study was approved by the Institutional Review Board at the University of Houston (Study 004480) and was conducted in accordance with the tenets of the Declaration of Helsinki.

### Protocol

**Fig. 1:**
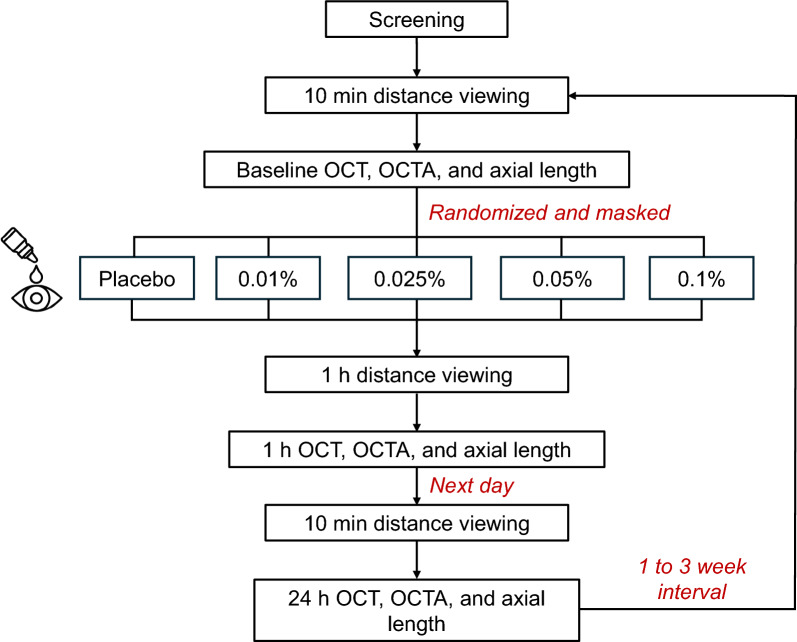
Study protocol

The protocol is shown in Fig. 1. Following screening, each participant was scheduled for five experimental conditions, which included instillation of one of four atropine concentrations, 0.01%, 0.025%, 0.05%, 0.1%, or a placebo (0.0%). Sterile ophthalmic atropine solutions (pH 4.5) and placebo (pH 5) were compounded locally (Greenpark Compounding Pharmacy, Houston, Texas, USA) and stored at 35 °F. The atropine solution was comprised of the appropriate dosage of atropine plus hypromellose, phosphate buffer, ethylene diamine tetra acetic acid, and water for irrigation. The placebo was the same solution, but without atropine. One team member (LAO) assigned codes to the bottles, and the investigator (BL) and participants were masked to the concentration used in each session. The same bottles (batch) of atropine were consistent across all participants. The concentrations were administered in a randomized order across five separate sessions; each spaced one to 3 weeks apart. All sessions commenced between 8:30 a.m and 11:30 a.m. to minimize the impact of diurnal variations. For each participant, all sessions began within a 1-h window. Participants were instructed not to wear contact lenses on the experiment days and to use spectacles, if necessary. Participants were asked to refrain from alcohol, caffeine, tobacco use, and vigorous physical activity on the experimental days to minimize effects on ocular physiology.

During each experimental session, measurements of the right eye were taken at three time points, baseline, 1 h and 24 h (± 1 h) after instilling a single drop of placebo or atropine solution. First, participants watched television at 6 m for 10 min to reduce the influence of prior activities on ocular structures. Then, baseline spectral-domain OCT and OCTA scans (Spectralis; Heidelberg Engineering, Heidelberg, Germany) were captured (described below), and axial length was measured (MYAH, VISIA, San Giovanni Valdarno, Italy). For axial length, three measurements (within ± 0.01 standard deviation) were averaged for each time point. Dynamic and static pupil and accommodation measurements were also recorded at each time point. Pupil and accommodation findings showing a dose dependent response have been previously published [22] and are not discussed further here. Following baseline measurements, a single drop of placebo or atropine solution was instilled. Participants continued to watch the distance television for 1 h, at which time all measurements were repeated. Participants then went about their usual daily schedule and returned to lab at 24 h for the final measurements.

**Fig. 2:**
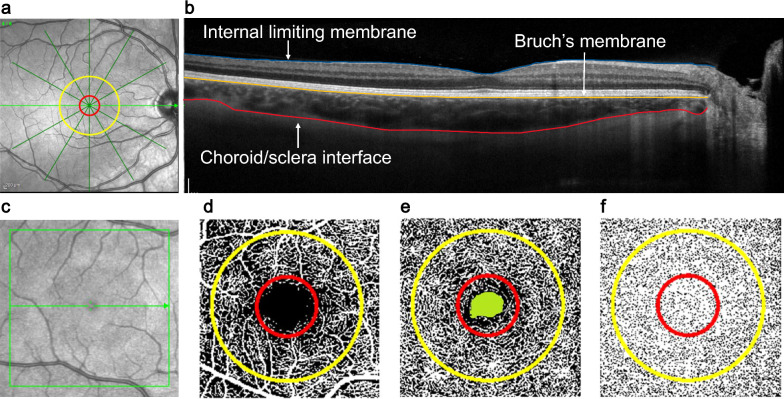
Optical coherence tomography (OCT) and optical coherence tomography angiography (OCTA) imaging. **a** 30° six-line radial OCT scan centered on the fovea (left), red ring indicates the central 1.0 mm diameter, and the yellow ring indicates the 1.0–3.0 mm annulus; **b** OCT b-scan with segmented boundaries, including the internal limiting membrane (blue), Bruch’s membrane (yellow), and choroid/sclera boundary (red); **c** 10° macular OCTA scan with representative binarized images showing the (**d**) superficial vascular complex, (**e**) deep vascular complex with foveal avascular zone (green), and (**f**) choriocapillaris; red ring indicates the central 1.0 mm diameter, and the yellow ring indicates the 1.0–2.5 mm annulus

For OCT, two high resolution enhanced depth six-line 30-degree radial scans centered on the fovea were captured while the participant fixated on the internal target. Scans with less than 30 dB signal strength were repeated. The 1 h and 24 h OCT scans were captured in follow-up mode. Raw OCT data (*.vol files) were exported and analyzed using a previously described custom automated segmentation algorithm in MATLAB (MathWorks, Natick, MA) to extract retinal and choroidal thickness. Scans for each participant were adjusted for lateral magnification [23–26]. The internal limiting membrane, retinal pigment epithelium, and choroid/scleral interface were segmented automatically and corrected manually, when necessary, by an experienced observer (BL)**,** masked to the atropine concentrations for which the scans were captured. The average retinal and choroidal thickness in the 1.0 mm diameter and 1.0–3.0 mm annulus centered at the fovea were calculated (Fig. 2). The mean ± standard deviation (95% limits of agreement) of the difference in retinal and choroidal thickness in central 1.0 mm for 20 participants between the two repeated measurements (baseline session of the first visit) were − 0.1 ± 1.4 µm (− 2.9 to 2.8 µm) and 1.3 ± 4.8 µm (− 8.2 to 10.8 µm), demonstrating high precision and reliability of the instrument.

For OCTA, one 10° × 10° macular scan was captured in enhanced depth high resolution mode (512 A-scans per B-scan, and 496 B-scans per volume). The scan was repeated if signal strength was less than 30 dB, if there were motion artifacts, or if the scan was not centered on the fovea [27]. The superficial vascular complex (between the internal limiting membrane and inner plexiform layer), deep vascular complex (between the inner nuclear layer and outer plexiform layer), and choriocapillaris (positioned beneath Bruch’s membrane, spanning from 10 to 30 µm) *en face* images were exported (.tiff format). The deep vascular complex and choriocapillaris were exported after projection artifact removal. The images were scaled for each participant’s individual lateral magnification using a previously described protocol [25, 26]. Images were analyzed using a custom image analysis MATLAB program and ImageJ to extract metrics including the foveal avascular zone (FAZ) area and perfusion density. The FAZ margin was marked on the magnification corrected deep vascular complex images using ImageJ by an experienced observer (BL), again masked to the atropine concentrations for which the scans were captured, and the FAZ area was extracted [28].

To extract perfusion density, the superficial and deep vascular complex images were binarized using the Otsu auto thresholding method [29]. The choriocapillaris was binarized using the local Phansalkar method [30]. Perfusion density was calculated as the ratio of area occupied by the blood vessels to the total measured area in the central 1.0 mm diameter and 1.0–2.5 mm annulus centered on the fovea of the binarized superficial vascular complex and deep vascular complex and choriocapillaris images (Fig. 2). Superficial and deep vascular complex and choriocapillaris perfusion densities in 1.0–2.5 mm annulus across the five baseline visits demonstrated good repeatability, with intraclass correlation coefficient values of 0.85, 0.76, and 0.79, respectively, and low measurement error (standard error of measurement = 0.021, 0.016, and 0.018, respectively).

Statistical analysis was performed using SPSS (version 27; IBM Corp, Armonk, NY) and Excel (version 2404; Microsoft Corporation, Redmond, WA). Descriptive statistics were calculated for axial length and all OCT and OCTA parameters and are provided as mean ± standard deviation, unless otherwise noted. Changes in each parameter at 1 h and 24 h were calculated. Two-way repeated measures analysis of variance (ANOVA) with Bonferroni-adjusted post hoc pairwise comparisons were used to assess changes in axial length and OCT and OCTA parameters across time points and concentrations. Assumptions of repeated measures ANOVA were evaluated prior to inference. Sphericity was assessed using Mauchly’s test, and Greenhouse–Geisser corrections were applied when violations were detected. Normality of errors was assessed by inspection of standardized residuals using Q-Q plots, supported by normality test results, and did not indicate substantial deviation from normality or the presence of influential outliers. Based on these observations, outcomes are summarized using mean ± standard deviation.

## Results

Twenty-one young adults were enrolled. One participant withdrew after experiencing prolonged pupil dilation that persisted for over a month following the first concentration of atropine; their data were excluded from the analysis. Upon unmasking at the end of the experiment, it was revealed that this participant’s first concentration was 0.1%. For all other participants, pupil diameter returned to baseline within the interval between sessions. The twenty participants included 10 females and 10 males with a mean age of 25.5 ± 3.4 years (range: 19–33 years). Mean spherical equivalent refraction of right eyes was − 1.91 ± 2.24 D (range: − 6.62 to + 0.75 D), including 12 myopes, 6 emmetropes, and 2 hyperopes.

**Fig. 3:**
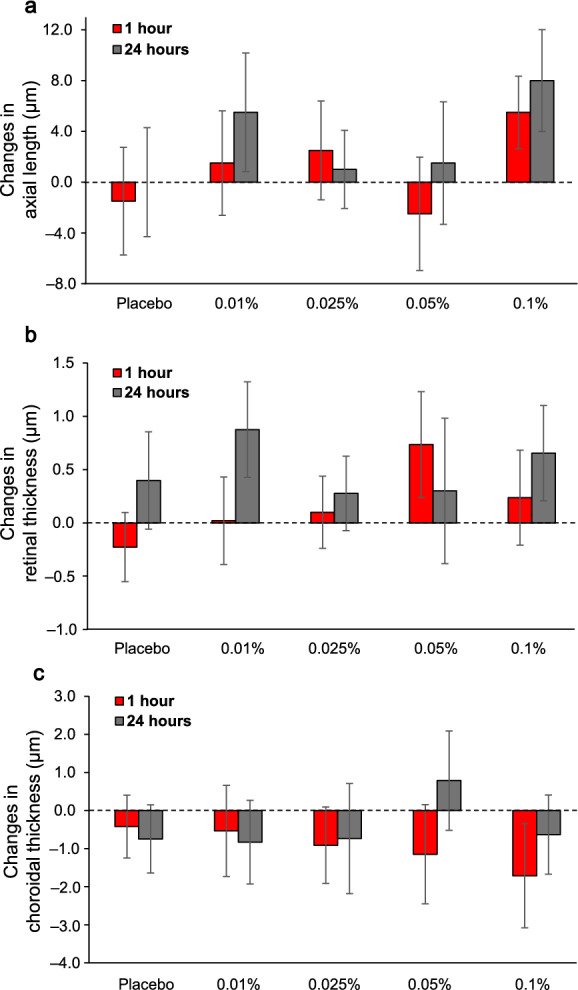
Mean change (± standard error of mean) from baseline in (**a**) axial length, (**b**) retinal thickness, and (**c**) choroidal thickness in the central 1.0 mm diameter at 1 h (red) and 24 h (grey) and across concentrations

Tables 1 and 2 summarize axial length and OCT and OCTA parameters for the three time points and five concentrations. Repeated measures ANOVA results for all outcome measures, including F values, degrees of freedom, and effect size estimates, are provided in Table 3. Figure 3 shows changes from baseline in axial length and retinal and choroidal thickness. There were no significant changes in axial length or retinal or choroidal thickness in the central 1.0 mm diameter or 1.0–3.0 mm annulus by time or atropine concentration, and no significant interaction between time and concentration (*P* > 0.05 for all).

**Table 1:** Axial length and optical coherence tomography (OCT) parameters [mean ± standard deviation (95% confidence interval)] for three time points (baseline, 1 h, and 24 h) and five atropine concentrations (placebo, 0.01%, 0.025%, 0.05%, and 0.1%)

Outcome measure		Concentration	Baseline	1 h	24 h
Axial length (mm)		Placebo	24.377 ± 1.255 (23.827–24.927)	24.375 ± 1.266 (23.820–24.930)	24.377 ± 1.267 (23.821–24.933)
		0.01%	24.377 ± 1.257 (23.826–24.928)	24.379 ± 1.257 (23.828–24.930)	24.383 ± 1.268 (23.827–24.939)
		0.025%	24.380 ± 1.261 (23.828–24.932)	24.383 ± 1.259 (23.832–24.934)	24.380 ± 1.264 (23.826–24.934)
		0.05%	24.381 ± 1.260 (23.829–24.933)	24.378 ± 1.259 (23.827–24.929)	24.382 ± 1.264 (23.828–24.936)
		0.10%	24.376 ± 1.264 (23.822–24.930)	24.382 ± 1.264 (23.828–24.936)	24.384 ± 1.262 (23.831–24.937)
Retinal thickness(µm)	Central 1.0 mm diameter	Placebo	264.81 ± 20.86 (255.80–274.20)	264.58 ± 20.80 (255.80–274.20)	265.21 ± 21.11 (255.80–274.20)
		0.01%	265.32 ± 20.80 (255.80–274.20)	265.32 ± 20.80 (255.80–274.20)	265.34 ± 21.08 (256.80–275.20)
		0.025%	265.52 ± 20.96 (256.80–275.20)	265.62 ± 20.83 (256.80–275.20)	265.80 ± 20.96 (256.80–275.20)
		0.05%	265.45 ± 21.00 (255.80–274.20)	266.18 ± 20.44 (257.24–274.76)	265.75 ± 21.89 (256.36–275.64)
		0.10%	266.22 ± 21.54 (256.36–275.64)	266.46 ± 21.26 (256.80–275.20)	266.89 ± 21.81 (257.36–276.64)
	Central 1.0–3.0 mm annulus	Placebo	336.79 ± 17.86 (329.12–344.88)	336.34 ± 17.59 (329.12–344.88)	337.21 ± 18.17 (329.12–344.88)
		0.01%	337.05 ± 18.12 (329.12–344.88)	336.34 ± 18.68 (327.68–344.32)	337.52 ± 18.48 (330.12–345.88)
		0.025%	336.90 ± 17.38 (329.55–344.45)	337.07 ± 17.73 (329.12–344.88)	337.17 ± 17.61 (329.12–344.88)
		0.05%	336.95 ± 17.76 (329.12–344.88)	336.95 ± 17.91 (329.12–344.88)	336.85 ± 18.18 (329.12–344.88)
		0.10%	337.48 ± 18.06 (329.12–344.88)	337.00 ± 17.89 (329.12–344.88)	337.67 ± 18.37 (330.12–345.88)
Choroidal thickness (µm)	Central 1.0 mm diameter	Placebo	366.90 ± 67.09 (337.65–396.35)	366.48 ± 66.27 (337.09–394.91)	366.15 ± 66.86 (336.65–395.35)
		0.01%	364.09 ± 67.38 (334.65–393.35)	363.55 ± 69.01 (333.78–394.22)	363.26 ± 67.57 (333.22–392.78)
		0.025%	365.55 ± 66.57 (336.65–395.35)	36.64 ± 65.36 (336.53–393.47)	364.81 ± 64.40 (336.97–393.03)
		0.05%	361.12 ± 68.68 (330.78–391.22)	359.98 ± 68.59 (329.78–390.22)	361.91 ± 68.47 (332.22–391.78)
		0.10%	362.86 ± 70.52 (331.90–394.10)	361 ± 71 (329.90–392.10)	362.23 ± 68.92 (331.78–392.22)
	Central 1.0–3.0 mm annulus	Placebo	354.62 ± 65.36 (326.53–383.47)	353.94 ± 64.53 (325.53–382.47)	354.64 ± 64.70 (326.53–383.47)
		0.01%	351.50 ± 66.15 (323.09–380.91)	351.34 ± 67.05 (321.65–380.35)	350.86 ± 65.31 (322.53–379.47)
		0.025%	353.00 ± 63.42 (325.41–380.59)	352.84 ± 63.94 (324.97–381.03)	352.96 ± 63.52 (324.97–381.03)
		0.05%	350.32 ± 67.77 (320.22–379.78)	349.04 ± 67.83 (319.22–378.78)	350.92 ± 67.04 (321.65–380.35)
		0.10%	350.66 ± 68.19 (321.22–380.78)	349.83 ± 67.61 (320.22–379.78)	350.57 ± 66.57 (321.65–380.35)

**Table 2:** Optical coherence tomography angiography (OCTA) parameters [mean ± standard deviation (95% confidence interval)] for three time points (baseline, 1 h, and 24 h) and five atropine concentrations (placebo, 0.01%, 0.025%, 0.05%, and 0.1%)

Outcome measure		Concentration	Baseline	1 h	24 h
Perfusion density of superficial vascular complex (%)	Central 1.0 mm diameter	Placebo	4.56 ± 2.39 (3.51–5.61)	4.62 ± 2.33 (3.60–5.64)	4.55 ± 2.76 (3.34–5.76)
		0.01%	4.97 ± 2.89 (3.70–6.24)	4.97 ± 3.26 (3.54–6.40)	4.94 ± 3.22 (3.53–6.35)
		0.025%	4.86 ± 2.90 (3.59–6.13)	4.90 ± 3.07 (3.56–6.24)	4.94 ± 3.09 (3.59–6.29)
		0.05%	5.27 ± 3.26 (3.84–6.70)	5.14 ± 3.13 (3.77–6.51)	4.86 ± 2.99 (3.55–6.17)
		0.10%	4.96 ± 2.85 (3.71–6.21)	4.90 ± 2.79 (3.68–6.12)	5.03 ± 2.89 (3.76–6.30)
	Central 1.0–2.5 mm annulus	Placebo	37.65 ± 6.09 (34.98–40.32)	36.90 ± 6.04 (34.25–39.55)	37.67 ± 5.88 (35.09–40.25)
		0.01%	38.03 ± 6.12 (35.35–40.71)	37.07 ± 6.09 (34.40–39.74)	38.12 ± 6.43 (35.30–40.94)
		0.025%	38.21 ± 6.04 (35.56–40.86)	38.22 ± 5.73 (35.71–40.73)	38.27 ± 5.99 (35.65–40.89)
		0.05%	38.42 ± 6.08 (35.76–41.08)	38.01 ± 6.58 (35.13–40.89)	37.94 ± 5.54 (35.51–40.37)
		0.10%	39.35 ± 6.33 (36.58–42.12)	36.98 ± 6.40 (34.18–39.78)	38.04 ± 6.38 (35.25–40.83)
Foveal avascular zone area (mm^2^)		Placebo	0.27 ± 0.11 (0.22–0.32)	0.27 ± 0.11 (0.22–0.32)	0.27 ± 0.11 (0.22–0.32)
		0.01%	0.28 ± 0.12 (0.23–0.33)	0.28 ± 0.12 (0.23–0.33)	0.28 ± 0.12 (0.23–0.33)
		0.025%	0.28 ± 0.12 (0.23–0.33)	0.28 ± 0.12 (0.23–0.33)	0.27 ± 0.12 (0.22–0.32)
		0.05%	0.27 ± 0.12 (0.22–0.32)	0.27 ± 0.12 (0.22–0.32)	0.27 ± 0.12 (0.22–0.32)
		0.10%	0.28 ± 0.12 (0.23–0.33)	0.28 ± 0.12 (0.23–0.33)	0.28 ± 0.12 (0.23–0.33)
Perfusion density of deep vascular complex (%)	Central 1.0 mm diameter	Placebo	16.07 ± 4.46 (14.12–18.02)	15.97 ± 4.36 (14.06–17.88)	15.98 ± 5.25 (13.68–18.28)
		0.01%	16.67 ± 5.03 (14.47–18.87)	16.97 ± 5.03 (14.77–19.17)	16.59 ± 5.50 (14.18–19.00)
		0.025%	17.06 ± 5.33 (14.73–19.39)	16.89 ± 5.22 (14.61–19.17)	16.54 ± 5.32 (14.21–18.87)
		0.05%	16.46 ± 5.43 (14.08–18.84)	16.77 ± 5.10 (14.54–19.00)	16.72 ± 4.74 (14.65–18.79)
		0.10%	16.79 ± 4.65 (14.75–18.83)	16.91 ± 4.82 (14.80–19.02)	17.09 ± 4.77 (15.00–19.18)
	Central 1.0–2.5 mm annulus	Placebo	43.91 ± 3.85 (42.22–45.60)	43.22 ± 3.24 (41.80–44.64)	43.80 ± 3.51 (42.26–45.34)
		0.01%	43.87 ± 3.43 (42.37–45.37)	43.89 ± 3.11 (42.53–45.25)	44.24 ± 3.42 (42.74–45.74)
		0.025%	43.15 ± 3.77 (41.50–44.80)	44.22 ± 3.57 (42.66–45.78)	44.15 ± 4.09 (42.36–45.94)
		0.05%	43.87 ± 4.34 (41.97–45.77)	43.63 ± 3.87 (41.94–45.32)	44.30 ± 3.39 (42.82–45.78)
		0.10%	44.11 ± 3.79 (42.45–45.77)	44.39 ± 4.45 (42.44–46.34)	44.19 ± 4.24 (42.33–46.05)
Perfusion density of choriocapillaris (%)	Central 1.0 mm diameter	Placebo	80.55 ± 6.33 (77.78–83.32)	81.57 ± 5.88 (79.00–84.14)	81.55 ± 7.14 (78.42–84.68)
		0.01%	81.41 ± 5.67 (78.93–83.89)	81.60 ± 6.19 (78.89–84.31)	81.95 ± 5.81 (79.41–84.50)
		0.025%	83.35 ± 4.57 (81.35–85.35)	84.33 ± 4.94 (82.17–86.49)	83.98 ± 4.28 (82.11–85.85)
		0.05%	81.37 ± 5.19 (79.10–83.64)	81.75 ± 6.20 (79.03–84.47)	82.86 ± 5.64 (80.39–85.33)
		0.10%	81.91 ± 6.76 (78.95–84.87)	83.66 ± 4.19 (81.83–85.49)	83.19 ± 4.71 (81.13–85.25)
	Central 1.0–2.5 mm annulus	Placebo	78.02 ± 4.76 (75.94–80.10)	78.07 ± 4.27 (76.20–79.94)	78.19 ± 4.76 (76.11–80.27)
		0.01%	77.95 ± 4.57 (75.95–79.95)	78.21 ± 4.27 (76.34–80.08)	78.01 ± 4.02 (76.25–79.77)
		0.025%	78.60 ± 3.67 (76.99–80.21)	79.20 ± 4.09 (77.41–80.99)	78.64 ± 3.73 (77.01–80.27)
		0.05%	78.04 ± 4.04 (76.27–79.81)	77.99 ± 4.03 (76.23–79.76)	78.65 ± 4.44 (76.70–80.60)
		0.10%	77.74 ± 4.94 (75.58–79.90)	78.75 ± 3.67 (77.14–80.36)	78.11 ± 3.82 (76.44–79.78)

**Table 3:** Repeated measures ANOVA summary for all the outcome measures

Outcome measure			Effect	F value	df1	df2	*P* value	Partial η^2^
Axial length (mm)			Concentration^#^	0.39	2.55	48.40	0.73	0.02
			Time	0.80	2.00	38.00	0.46	0.04
			Time × Concentration	0.69	8.00	152.00	0.70	0.03
Retinal thickness (µm)	Central 1.0 mm diameter		Concentration^#^	2.58	2.29	43.51	0.08	0.12
			Time	1.78	2.00	38.00	0.18	0.09
			Time × Concentration	0.73	8.00	152.00	0.67	0.04
	Central 1.0–3.0 mm annulus		Concentration	0.55	4.00	76.00	0.70	0.03
			Time	1.62	2.00	38.00	0.21	0.08
			Time × Concentration^#^	0.63	3.61	68.54	0.63	0.03
Choroidal thickness (µm)	Central 1.0 mm diameter		Concentration	0.95	4.00	76.00	0.44	0.05
			Time	1.02	2.00	38.00	0.37	0.05
			Time × Concentration	0.39	8.00	152.00	0.92	0.02
	Central 1.0–3.0 mm annulus		Concentration	0.74	4.00	76.00	0.57	0.04
			Time	0.39	2.00	38.00	0.68	0.02
			Time × Concentration	0.23	8.00	152.00	0.98	0.01
Perfusion density of superficial vascular complex (%)	Central 1.0 mm diameter		Concentration^#^	0.96	1.77	35.56	0.38	0.05
			Time	0.25	2.00	38.00	0.78	0.01
			Time × Concentration	0.39	8.00	152.00	0.92	0.02
	Central 1.0–2.5 mm annulus		Concentration	0.65	4.00	76.00	0.63	0.03
			Time	4.52	2.00	38.00	0.02	0.21
			Time × Concentration	1.06	8.00	152.00	0.39	0.05
Foveal avascular zone area (mm^2^)			Concentration^#^	0.76	2.67	50.75	0.51	0.04
			Time^#^	0.75	1.38	26.13	0.43	0.04
			Time × Concentration^#^	1.09	1.88	34.74	0.34	0.05
Perfusion density of deep vascular complex (%)	Central 1.0 mm diameter		Concentration^#^	2.73	2.87	43.17	0.06	0.13
			Time	0.25	2.00	38.00	0.79	0.01
			Time × Concentration	0.37	8.00	152.00	0.93	0.02
	Central 1.0–2.5 mm annulus		Concentration	0.75	4.00	76.00	0.56	0.04
			Time	1.47	2.00	38.00	0.32	0.07
			Time × Concentration	0.51	8.00	152.00	0.85	0.03
Perfusion density of choriocapillaris (%)	Central 1.0 mm diameter		Concentration	2.49	4.00	76.00	0.06	0.12
			Time	1.64	2.00	38.00	0.21	0.08
			Time × Concentration	0.19	8.00	152.00	0.99	0.01
	Central 1.0–2.5 mm annulus		Concentration	1.15	4.00	76.00	0.34	0.06
			Time	1.39	2.00	38.00	0.26	0.09
			Time × Concentration	0.69	8.00	152.00	0.70	0.04

Sphericity was assessed using Mauchly’s test. When violations were detected (*P* < 0.05), Greenhouse–Geisser-corrected degrees of freedom (df) are reported and indicated by #

Visual inspection of standardized residuals for each outcome measure using Q-Q plots, supported by normality test results (*P* > 0.05), indicated no significant deviation from normality

**Fig. 4:**
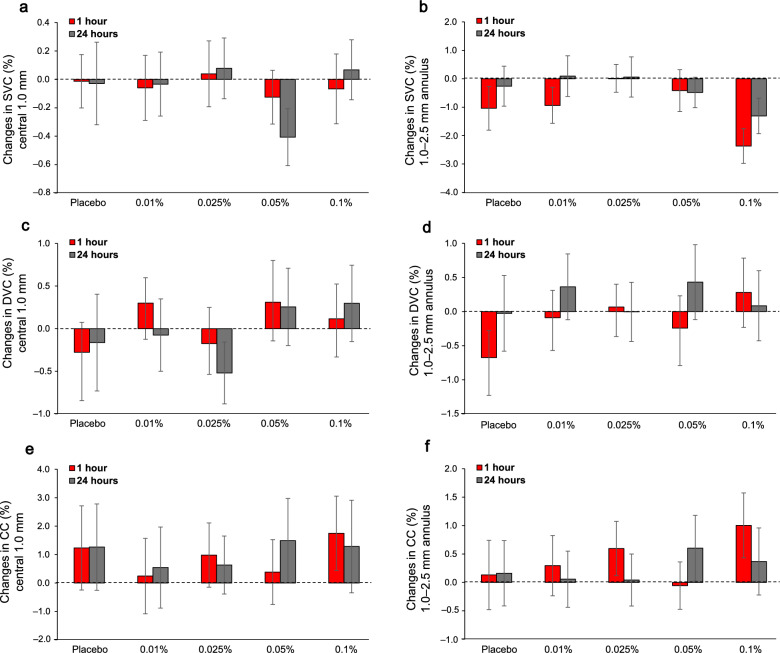
Mean change (± standard error of mean) from baseline in perfusion density of the (**a**, **b**) superficial vascular complex (SVC), (**c**, **d**) deep vascular complex (DVC), and (**e**, **f**) choriocapillaris (CC) in central 1 mm diameter (left column) and 1.0–2.5 mm annulus (right column) at 1 h (red) and 24 h (grey) and across concentrations

Figure 4 shows changes from baseline in OCTA parameters. For perfusion density of the superficial and deep vascular complex in the central 1.0 mm diameter centered on the fovea, there were no significant changes across time or atropine concentration and no significant interaction between time and concentration (*P* > 0.05 for all). For the 1.0–2.5 mm annulus, perfusion density of the superficial vascular complex decreased (*P* = 0.02) over time after atropine instillation, but not concentration (*P* > 0.05). Upon post hoc analysis across time, a significant decrease in the perfusion density compared to baseline was found at 1 h (− 1.3% ± 1.2%, *P* = 0.03), but not at 24 h (− 0.4% ± 0.6%, *P* = 0.28). No interaction between concentration and time was noted in 1.0–2.5 mm annulus perfusion density of the superficial vascular complex. There were no significant changes in perfusion density of the deep vascular complex or choriocapillaris in 1.0–2.5 mm annulus after atropine (*P* > 0.05). The FAZ area remained unchanged after atropine (*P* > 0.05).

## Discussion

This study investigated axial length, retinal and choroidal thickness, and microvasculature metrics 1 h and 24 h after topical instillation of a range of low concentration atropine concentrations in healthy adults using a double masked repeated measures study design. Findings showed that a single drop of 0.01%, 0.025%, 0.05% and 0.1% atropine does not significantly affect axial length or retinal or choroidal thickness after 1 h or 24 h. However, atropine induced transient changes in perfusion density of the superficial vascular complex that was time dependent but not concentration dependent, as observed with OCT angiography.

Early clinical trials in children utilized 1% atropine, which showed significant efficacy in reducing myopia progression and axial elongation [31]. With 1% atropine treatment, choroidal thickening was observed within 1 week, with effects maintained for 6 months [20]. Adverse effects such as photophobia and blurring of near vision limit 1% atropine use, prompting interest in low concentration atropine (≤ 0.1%), which effectively slows myopia with fewer side effects [14]. A meta-analysis suggested that 0.05% atropine may be the optimal concentration providing a balance between efficacy and risk of adverse events [14].

The choroid is an extensively vascularized tissue that supplies oxygen and nutrients to the outer retina and sclera [32]. Previous studies have shown that the choroid receives dense autonomic innervation, involving both adrenergic and muscarinic receptor activation [33]. Several animal studies have shown that non-selective or partially selective muscarinic antagonists can significantly influence choroidal thickness, as well as ocular growth [34–36]. In humans, studies investigating the short-term effects of topical anticholinergic agents, including atropine and homatropine, on choroidal thickness have found inconsistent results [19, 37, 38].

Axial length was included in the current study to aid in interpreting choroidal thickness changes after atropine. Axial length is measured from the cornea to the retinal pigment epithelium. Therefore, transient changes in axial length are complementary to changes in choroidal thickness and often used as a surrogate measure of choroidal thickness change in previous studies [17, 31]. Here, axial length and choroidal thickness did not change significantly at 1 h or 24 h for any of the concentrations tested. This is in contrast to a previous study, which reported a 6 µm decrease in axial length and 6 µm increase in subfoveal choroidal thickness 60 min after instillation of a single dose of 0.01% atropine in young healthy myopic adults [39]. Chiang and Philip reported no changes in choroidal thickness 22 h after instillation of 0.5% atropine in young adults [40]. These findings indicate that short-term choroidal changes following atropine are inconsistent across studies.

Given that no changes in axial length or choroidal thickness were observed in our study, it raises the question of whether a single instillation effectively penetrated the ocular tissue. However, as part of this same study in the same participants, our previously published findings showed that pupil and accommodation metrics were significantly altered at 1 h and 24 h in a dose dependent manner, indicating significant penetration into the eye [22]. Additionally, a previous study showed that a single drop of 0.01% atropine was detectable in both the aqueous and vitreous humor 6 h after instillation [41]. The aqueous and vitreous humor were sampled from patients undergoing cataract surgery and vitreoretinal surgery for epiretinal membrane/macular hole, respectively. In porcine eyes, aqueous humor concentration increased in a dose-dependent manner following administration of 0.01%, 0.1%, and 0.5% atropine [42]. Similarly, in rabbits, atropine was detected in ocular tissues 5 h post-instillation, with distribution shifting to posterior tissues at 24 h, showing higher levels in the posterior sclera compared to the retina [43].

It is plausible that short-term effects of atropine on choroidal thickness differ from long-term effects. Daily atropine administration may lead to cumulative ocular absorption, producing choroidal changes that differ from those observed following a single instillation. Interestingly, a study in children observed choroidal thickening following daily 0.01% atropine use for 1 week and 3 months, but choroidal thinning after 6 months of continued treatment [20].

Although atropine is widely used to slow myopia progression, its underlying mechanisms remain unclear. The retina has been proposed as a potential site of action. In chicks, atropine was shown to activate retinal cells in a non-specific manner, which may lead to dopamine release, thereby suppressing myopia [44]. In humans, a study using 0.1% atropine suggested that amacrine cells may mediate its action at the retinal level in myopia control [45]. Atropine may help re-establish neurotransmitter balance in the retina, which is typically altered during myopia progression [46]. In the present study, no significant changes in retinal thickness were observed over 24 h after instillation of different atropine concentrations. Similarly, a previous study found that retinal thickness was not affected by 0.01% atropine instilled daily for 1 month [21]. However, the absence of structural changes in retinal thickness does not rule out the possibility that the retina is a site of atropine’s action.

Assessment of the retinal and choroidal microvasculature using OCTA may offer additional insight into atropine’s mechanisms in myopia control. It has been suggested that atropine may increase nitric oxide, leading to choroidal vessel dilation and improved blood flow, mitigating hypoxic conditions in the sclera [32, 47, 48]. Findings on perfusion density following 3–6 months of 0.01% atropine are mixed, with some studies showing an increase and others showing no change [21, 49]. For example, a study in children found a transient increase in perfusion density in the foveal region in the initial 3 months of 0.125% atropine treatment [50]. Wang et al. reported that the choriocapillaris was not affected daily use of 0.01% atropine for 1–3 months [21]. The present study found a significant decrease in the perfusion density of the superficial vascular complex in the 1.0–2.5 mm region at 1 h after drop instillation, while the deep vascular layer and choriocapillaris remained unchanged. This transient reduction may reflect a redistribution of blood flow secondary to atropine induced increase in blood flow volume [48]. The localized atropine effect in the 1.0–2.5 mm annulus of the superficial vascular complex may reflect the higher proportion of medium-sized and larger vessels in this region, whereas the deep vascular complex and choriocapillaris are composed mainly of fine capillaries. Although no dose dependent effect emerged, the largest decrease in perfusion density of the superficial vascular complex and increase in perfusion density of choriocapillaris occurred for the highest (0.1%) atropine concentration. In guinea pigs, 1% atropine has been shown to enhance entire choroidal microcirculation [48]. Here, the FAZ remained unchanged after a single instillation. In contrast, a previous study found that FAZ area significantly decreased after 1 month of daily 0.125% atropine use [50]. Our study did not assess total choroidal perfusion. Future studies could evaluate atropine dose-dependent effects on choroidal blood flow with laser speckle flowgraphy for a more comprehensive assessment of choroidal effects.

A strength of the current study was that multiple low concentration atropine concentrations were examined in the same participants. While previous studies have investigated choroidal thickness and axial length after low concentration atropine [20, 51], they tested only a single atropine concentration (0.01% or 1%) or tested multiple concentrations, but in different participants. Other strengths were the double masked study design, tightly controlled imaging protocol, and automatic OCT segmentation methods.

Limitations of the current study include the following. First, only one instillation of atropine was investigated; however, this was for the purpose of understanding acute effects of atropine. The relatively small sample size may have limited power to identify minor effects in the present study, and the findings should be interpreted with caution. Future studies should consider investigating the cumulative effects of daily instillation. Second, only young adults were included. Although choroid morphology is generally similar between adults and children, myopia control is most applicable to myopic children, so future research should include myopic children of varying ages. High myopes were not included, so as to avoid participants with potential myopia-related retinal changes. However, there is no evidence that atropine would have differing effects in this group, though it could be explored in future studies.

## Conclusion

A single instillation of 0.01%, 0.025%, 0.05%, or 0.1% atropine did not result in detectable changes in axial length or retinal or choroidal thickness after 1 h or 24 h in young adults. However, atropine instillation was associated with a time-dependent transient reduction in perfusion density of the superficial vascular complex. Together, these findings suggest that short-term effects of atropine on axial length and retinal and choroidal parameters may differ from long-term effects.

## Data Availability

The datasets used and/or analyzed during the current study are available from the corresponding author on reasonable request.

## Abbreviations

OCT: Optical coherence tomography
OCTA: Optical coherence tomography angiography
FAZ: Foveal avascular zone
ANOVA: Analysis of variance
